# Longitudinal relations between sleep quality, psychological distress, and cognitive function among rural older adults: a cross-lagged network analysis

**DOI:** 10.1017/S0033291726105182

**Published:** 2026-07-28

**Authors:** Xin Che, Shujun Chai, Dan Zhao, Wanchen Wang, Chengchao Zhou

**Affiliations:** 1 Department of Social Medicine and Health Management, School of Public Health, Advanced Medical Research Institute, Cheeloo College of Medicine, Shandong University, Jinan, China; 2 NHC Key Lab of Health Economics and Policy Research (Shandong University), Jinan, China; 3 Center for Health Management and Policy Research, Shandong University (Shandong Provincial Key New Think Tank), Jinan, China; 4 School of Population and Health, Renmin University of China, Beijing, China; 5 School of Public Health, Xuzhou Medical University, Jiangsu, China; 6 Institute of Health and Elderly Care, Shandong University, Jinan, China

**Keywords:** cognitive function, cross-lagged panel network analysis, psychological distress, rural older adults, sleep quality

## Abstract

**Background:**

Sleep disorder, cognitive impairment, and psychological distress usually co-occur in older adults, yet their dynamic relationships over time remain poorly understood, particularly in rural aging populations.

**Methods:**

This study employed cross-lagged panel network (CLPN) analysis to examine temporal relationships among sleep quality, cognitive function, and psychological distress in 2,196 rural Chinese older adults across 4 years. We analyzed autoregressive and cross-lagged effects, centrality measures, and sex differences in network structures over two time intervals.

**Results:**

CLPN analysis revealed temporal associations among sleep quality, psychological distress, and cognitive function domains. Subjective sleep quality emerged as the most influential predictor node across both time periods. Cognitive components – particularly orientation, attention and calculation, and language – were identified as the most vulnerable target nodes. Sex-stratified analyses revealed moderate structural similarity between male and female networks across time periods.

**Conclusions:**

This study provides new evidence for understanding the temporal relationships among sleep quality, psychological distress, and cognitive function in rural older adults. These findings may suggest that subjective sleep quality represents a central and modifiable target within the aging-related symptom network, with potential implications for early intervention across cognitive and psychological domains.

## Introduction

With the accelerating pace of global aging, cognitive impairment has emerged as a critical public health challenge, especially in China, where the older adult population is growing at an unprecedented rate (The Lancet, 2022). Cognitive function encompasses multiple domains – such as memory, attention, executive function, language, and processing speed (Harvey, 2019) – which are intrinsically interconnected and generally decline with advancing age (Murman, 2015). Epidemiological studies highlight a substantial burden of cognitive impairment among older adults in China: mild cognitive impairment (MCI) and dementia affect an estimated 15.5% and 6% of the population aged 60 and older, respectively (Jia et al., 2020). The number of people living with dementia is projected to 48.98 million by 2050 (Li, Qin, Zhu, & Jia, 2021). Cognitive decline imposes a multifaceted burden encompassing not only direct healthcare costs but also substantial informal caregiving demands, reduced quality of life, and profound psychosocial effects on both patients and their families (Gläser et al., 2025; Pérez Palmer, Trejo Ortega, & Joshi, 2022; Wimo et al., 2023). Notably, rural older adults experience a higher prevalence of cognitive decline and impose additional burden on family caregivers compared to their urban counterparts (Jia et al., 2020; Wiese et al., 2023). Previous studies have identified several risk factors for cognitive impairment, including female sex, depressive symptoms, and poor sleep quality (Jia et al., 2021; Li, Wang, & Dupre, 2022).

Sleep disorders, psychological distress, and cognitive impairment frequently co-occur in older adults. Sleep disorders are common in this population and typically present as altered sleep architecture, reduced sleep duration, and fragmented sleep patterns (Pavlova & Latreille, 2019; Yaremchuk, 2018). Poor sleep quality has been linked to increased risk of neurodegenerative processes and cognitive decline (Casagrande, Forte, Favieri, & Corbo, 2022; Leng et al., 2020). Liu et al. reported that both sleep initiation difficulties and daytime sleepiness increased the risk of MCI, with daytime sleepiness further linked to a higher risk of dementia (Liu et al., 2025). Similarly, psychological distress, especially symptoms of depression and anxiety, is prevalent among older adults and often accelerates cognitive deterioration (Sutin, Stephan, & Terracciano, 2018). Previous studies have demonstrated bidirectional associations between sleep quality and psychological distress (Alimoradi et al., 2021; Madrid-Valero et al., 2023). Moreover, the complex interplay among sleep, psychological distress, and cognitive function has been supported by evidence suggesting that impaired sleep and heightened psychological distress synergistically contribute to cognitive impairment (Kim, Park, Zhu, & Fritschi, 2021). Despite these findings, much of the existing literature relies on cross-sectional designs or short-term follow-ups, limiting insights into temporal and causal dynamics, particularly in under-researched rural populations.

*Scarring theory* posits that adverse experiences can leave lasting ‘*scars*’ on an individual’s psychological and physiological well-being, leading to long-term functional impairment (Allemand, Grünenfelder-Steiger, & Flückiger, 2018). Over time, psychological issues may not only affect current cognitive function but can also exert persistent effects on future cognitive abilities (Farina et al., 2020; Gulpers et al., 2019). Moreover, sleep alterations have been recently conceptualized as biological ‘scars’ of depression (Palagini et al., 2013). In parallel, *vulnerability models* theorize those impairments in cognitive functioning (e.g. executive functioning deficits) act as predisposing factors that heighten susceptibility to psychological difficulties like fear and worry. For instance, Zainal et al. found that difficulties in working memory and inductive reasoning predict increased levels of uncontrollable worry (Zainal & Newman, 2018). Taken together, these two theoretical frameworks generate distinct directional predictions. Determining which predominates requires longitudinal examination of symptom temporal ordering to identify primary drivers and intervention targets.

Traditional studies have largely focused on syndrome-level associations, often relying on total scores of neuropsychological symptoms (Zainal & Newman, 2023). However, such aggregate measures may obscure meaningful interactions between individual symptoms and functional domains. According to the network theory of psychopathology (Borsboom, 2017), psychiatric disorders are not latent entities that cause symptoms, but rather systems of causally interconnected symptoms that mutually reinforce one another. In this framework, sleep disturbances, psychological distress, and cognitive function are interconnected. Importantly, symptoms across different disorders can reciprocally influence one another, creating vicious cycles that contribute to the development and maintenance of comorbid conditions (Cramer, Waldorp, van der Maas, & Borsboom, 2010). The strength and influence of these symptom relationships can be quantified through centrality indices and edge weights. Centrality measures help identify which symptoms act as key drivers of network-wide activation (Robinaugh, Millner, & McNally, 2016). Consequently, pinpointing central and bridging symptoms within such networks offers critical insights into the mechanisms underlying comorbid disorders (Fried & Cramer, 2017).

Recently, network analysis methods have seen growing application in psychiatric and psychological research involving older adults. For instance, Lin et al. identified core and bridge symptoms linking emergent neuropsychiatric symptoms and sleep problems (Lin et al., 2025). Bai explored bridge symptoms – specifically ‘Naming’ and ‘Language’ – between depressive symptoms and cognitive performance, noting that improved sleep quality may enhance quality of life in older adults (Bai et al., 2023). However, a major limitation of prior network-based studies lies in their predominant use of cross-sectional designs, which offer limited evidence for causality (Bai et al., 2023). Cross-lagged panel network (CLPN) analysis combines cross-lagged panel models (CLPMs) with network analytic techniques, enabling a nuanced examination of how changes in one symptom at an earlier time point predict subsequent changes in others (Rhemtulla, Wysocki, Bork, & Cramer, 2022). This method helps uncover potential directional influences and feedback loops among symptoms. By clarifying temporal relationships while controlling for prior symptom levels and confounding factors, longitudinal data can strengthen causal inference (McNally, 2021) and provide a more detailed understanding of dynamic symptom interactions over time.

Emerging evidence indicates that the relationship between sleep quality, psychological distress, and cognitive function may differ by sex among older adults. Research has shown that males and females often exhibit different patterns of sleep disturbances: females are more likely to report insomnia and sleep quality complaints (Madrid-Valero et al., 2017; Zhang & Wing, 2006), whereas males show a higher prevalence of sleep-disordered breathing (Peppard et al., 2013). Similarly, well-documented sex differences exist in the manifestation and prevalence of psychological distress. Females generally report higher rates of depression and anxiety (Albert, 2015; Salk, Hyde, & Abramson, 2017), while males may often exhibit different symptom profiles and help-seeking behaviors (Seidler et al., 2016). These disparities may also extend to cognitive domains, where sex-specific vulnerabilities and protective factors could shape the trajectories of cognitive decline (Hajali, Andersen, Negah, & Sheibani, 2019; Podcasy & Epperson, 2016). Given established sex differences, it is crucial to examine whether the network structure and symptom interactions differ between males and females. Sex-stratified network analysis can reveal sex-specific pathways between sleep disturbances, psychological distress, and cognitive impairment. This approach can help to identify targeted intervention strategies and inform tailored approaches for promoting cognitive health in rural older adults.

This study aims to employ a longitudinal cross-lagged network approach to elucidate the reciprocal relationships among sleep quality, psychological distress, and cognitive function in Chinese rural older adults. By identifying key pathways and temporal dynamics, this research seeks to inform targeted, timely interventions to maintain cognitive health and improve overall well-being within this vulnerable population.

## Methods

### Participants and study design

This longitudinal study utilized data from the Shandong Rural Elderly Health Cohort (SREHC), which is an ongoing observational, prospective cohort investigating rural older adults aged over 60 years living in Shandong Province. The cohort study commenced in 2019 (T1), with subsequent follow-up assessments conducted in 2020 (T2) and 2022 (T3). A total of 2,395 participants completed all three waves of data collection. Of these, 199 individuals were excluded due to lack of complete data on at least one key variable (i.e., failed to complete an entire scale at one or more time points). Consequently, 2,196 participants with fully observed data on all key variables across all three waves were included in the final analysis.

#### Participant recruitment and selection

Multistage stratified random sampling method was used to select participants. Details of the study methodology are described in detail elsewhere (Wang et al., 2021; Yuan et al., 2022). Exclusion criteria included: (1) individuals with neurodegenerative disorders (e.g., dementia, Parkinson’s disease) or severe psychiatric conditions (e.g., schizophrenia, bipolar disorder); (2) participants with hearing or speech impairments preventing normal interpersonal communication and (3) individuals who refused to participate in the questionnaire survey.

#### Data collection

Trained interviewers conducted face-to-face assessments using standardized protocols. To minimize seasonal bias, all surveys were administered during the same 4-month window (May–August) in each wave. The SREHC survey covers multiple aspects of life among older adults, including demographics (i.e., age, sex, marital status, educational level), behavioral factors (smoking and drinking habits), individual physical and psychosocial health, cognitive function, and so on.

### Ethical approval and informed consent

Ethical approval was obtained from the Ethics Committee of Shandong University (Approval No. 201812328). Prior to participation, all individuals provided written informed consent after receiving a detailed explanation of the study procedures, potential risks, and benefits. Participants were assured of confidentiality and their right to withdraw from the study at any time without consequence.

### Measures

#### Sleep quality

Sleep quality symptoms were assessed using the Pittsburgh Sleep Quality Index (PSQI) (Buysse et al., 1989), a validated 19-item self-report questionnaire widely used in older adult populations (Qiu et al., 2016). The PSQI measures seven dimensions of sleep over the past month: subjective sleep quality, sleep latency, sleep duration, sleep efficiency, sleep disturbances, use of sleep medication, and daytime dysfunction. Each dimension is scored from 0 (no difficulty) to 3 (severe difficulty). The total score ranges from 0 to 21, with higher scores indicating poorer sleep quality. The Cronbach’s alpha coefficients for the PSQI were 0.765 (T1), 0.746 (T2), and 0.747 (T3).

#### Psychological distress

Psychological distress was measured using the Kessler Psychological Distress Scale (K10), a 10-item screening tool assessing nonspecific symptoms of anxiety and depression over the past 4 weeks (Kessler et al., 2002; Zhou et al., 2008). Responses are rated on a 5-point Likert scale (1 = ‘none of the time’ to 5 = ‘all of the time’). The total score ranges from 10 to 50, with higher scores indicating greater psychological distress. The Cronbach’s alpha coefficients for this scale were 0.902 (T1), 0.911 (T2), and 0.905 (T3).

#### Cognitive function

Cognitive function was assessed using the Mini-Mental State Examination (MMSE), a widely used 30-item screening tool adapted for older adults with varying education levels (Pangman, Sloan, & Guse, 2000). The MMSE evaluates six cognitive domains (Truong et al., 2024): orientation, registration, attention, recall, language, and visuospatial. Individual domain scores were calculated by summing responses to relevant items, with maximum scores varying by domain (detailed information is presented in Supplementary Table S1). The total score ranges from 0 to 30, with higher scores indicating better cognitive function. The Cronbach’s alpha coefficients for the questionnaire were 0.717 (T1), 0.761 (T2), and 0.760 (T3).

### Statistical analysis

Initial data cleaning and descriptive statistical analyses were performed using Stata MP 17.0 (Stata Corp, College Station, TX, USA). The longitudinal relationships between sleep quality, psychological distress, and cognitive function were examined using a CLPN analysis across three time points (T1, T2, and T3). The analysis was conducted using R version 4.4.2, following established methodological guidelines for CLPN modeling (Rhemtulla et al., 2022). The analysis controlled for covariates including age, sex, marital status, and educational level at T1 (in T1–T2 network) and T2 (in T2–T3 network).

The CLPN analysis was implemented using regularized regressions to estimate the complex relationships between variables across three time points. The estimation process focused on calculating both autoregressive effects (coefficients of a variable at T1/T2 predicting itself at T2/T3) and cross-lagged effects (coefficients of a variable at T1/T2 predicting different variables at T2/T3). Key R packages were instrumental in this process: *glmnet* for calculating regularized regressions (Friedman, Hastie, & Tibshirani, 2010), *qgraph* for network visualization (Epskamp et al., 2012), and *bootnet* for assessing network stability and accuracy (Epskamp, Borsboom, & Fried, 2018). The coefficients of logistic regressions (edge weights) were converted to odds ratios to facilitate interpretability, with edge weights smaller than 1 reflecting negative relations and edge weights greater than 1 denoting positive relations.

The analysis calculated two critical centrality indices for each variable. The cross-lagged ‘out’ Expected Influence (Out-EI) quantified the extent to which a variable at T1/T2 predicts other variables at T2/T3, while the cross-lagged ‘in’ Expected Influence (In-EI) measured the degree to which a variable at T2/T3 is predicted by other variables at T1/T2. Additionally, Bridge Expected Influence (BEI) was calculated to identify variables that serve as a bridge between different symptom domains. The 1-step BEI of a given node refers to the sum of the weights of the edges from this node to nodes in other communities, reflecting the extent and nature of the node’s direct influence on other domains.

To ensure the robustness of the network analysis, comprehensive bootstrapping approaches were employed. Nonparametric bootstrapping with 1000 iterations was used to compute 95% confidence intervals for each edge weight, providing a rigorous assessment of the statistical precision of network connections. Network stability was estimated using case-drop bootstrapping to calculate Correlation Stability (CS) coefficients, with coefficients >0.25 considered acceptable and those >0.50 deemed excellent.

Sex-based comparisons were conducted following established CLPN group comparison guidelines. This involved estimating (1) the correlation between edge lists, (2) the cumulative overlapping associations, and (3) the Jaccard Similarity Index, which is the proportion of edges present in both networks that also share the same edge features (direction and sign).

## Results

### Descriptive statistics

A total of 2,196 rural older adults were identified. The mean age of the respondents was 70.01 years (SD = 5.89) at T1, and 34.11% were male. The means and standard deviations of the study’s variables for the three waves, as well as the correlations between all study variables, are presented in Table 1. Additionally, the node labels and descriptive statistics are presented in Supplementary Table S2.

**Table 1.** Descriptive statistics and correlations for the main variables Table 1. long description.

Variables	Mean	Standard deviation	Sex	Education level	T1 marital status	T1 age	T1 PSQI	T2 PSQI	T3 PSQI	T1 K10	T2 K10	T3 K10	T1 MMSE	T2 MMSE
Sex	1.66	0.47	–											
Education level	1.24	0.53	−0.250***	–										
T1 marital status	0.76	0.43	−0.129***	0.137***	–									
T1 age	70.01	5.89	−0.055**	−0.158***	−0.257***	–								
T1 PSQI	7.68	4.36	0.227***	−0.105***	−0.063**	−0.021	–							
T2 PSQI	8.86	4.17	0.261***	−0.120***	−0.071***	0.016	0.683***	–						
T3 PSQI	6.59	4.15	0.242***	−0.100***	−0.039	0.015	0.448***	0.499***	–					
T1 K10	16.43	7.27	0.150***	−0.078***	−0.002	−0.035	0.462***	0.376***	0.271***	–				
T2 K10	17.92	7.88	0.156***	−0.100***	−0.039	−0.013	0.392***	0.426***	0.288***	0.694***	–			
T3 K10	15.66	6.75	0.098***	−0.125***	−0.050*	0.029	0.237***	0.277***	0.382***	0.285***	0.345***	–		
T1 MMSE	23.17	4.92	−0.267***	0.314***	0.220***	−0.191***	−0.117***	−0.138***	−0.131***	−0.158***	−0.180***	−0.165***	–	
T2 MMSE	23.46	5.40	−0.239***	0.299***	0.218***	−0.216***	−0.111***	−0.136***	−0.127***	−0.107***	−0.149***	−0.168***	0.778***	–
T3 MMSE	23.21	5.23	−0.239***	0.311***	0.221***	−0.248***	−0.065**	−0.096***	−0.104***	−0.084***	−0.122***	−0.206***	0.665***	0.699***

*Note:* Sex was coded as 1 = male, 2 = female.

The educational attainment was classified into three categories: illiterate or elementary school (1), junior high school (2), and high school or above (3).

The marital status was divided into two categories: single (0) and married (1), of which single includes those who are unmarried, divorced and widowed.

*p < 0.05; **p < 0.01; ***p < 0.001.

### CLPN results

**Figure 1.**
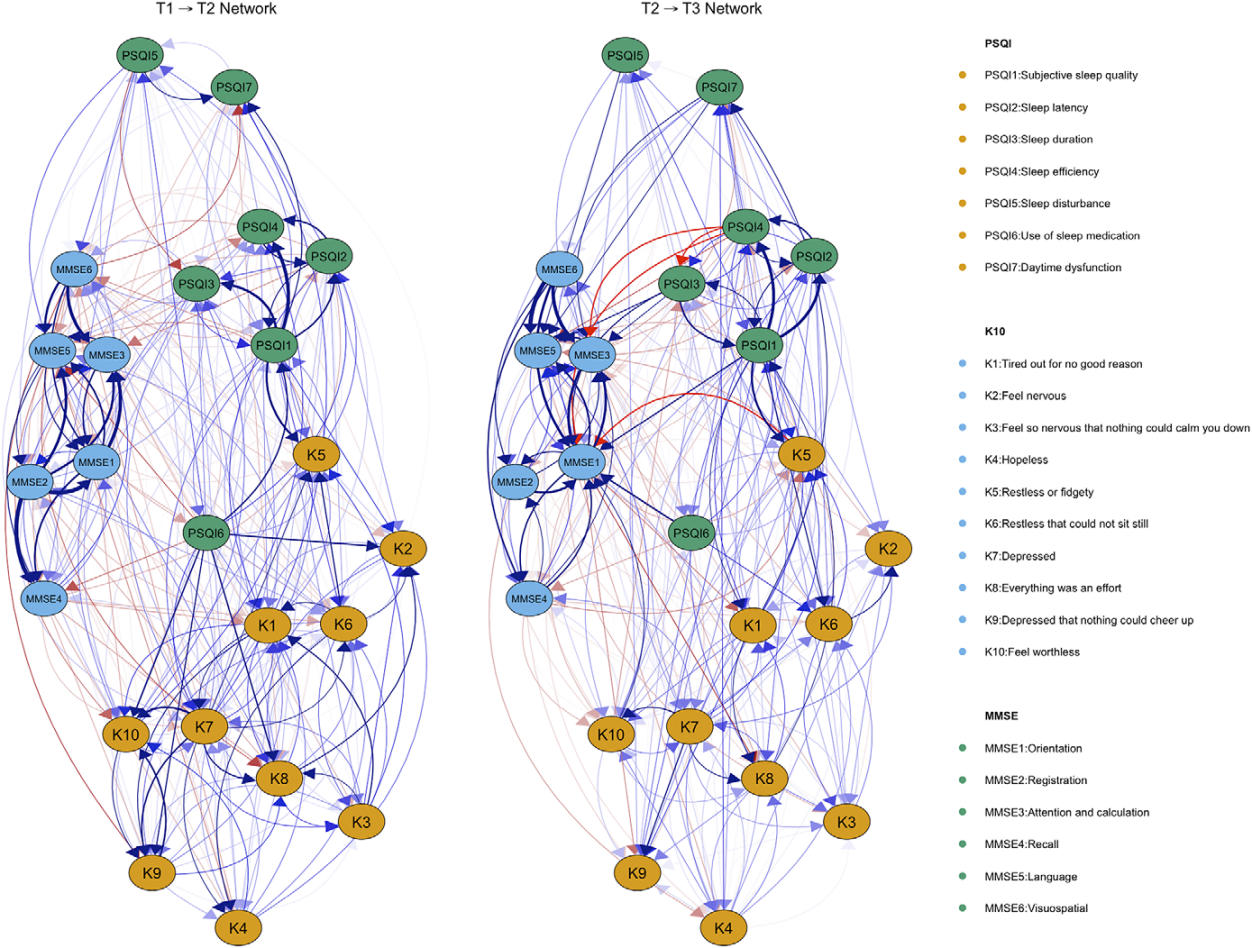
Cross-lagged panel network of sleep quality, psychological distress, and cognitive function. *Note: N* = 2,196. Blue arrows indicate positive coefficients and red arrows indicate negative coefficients. Arrow thickness represents the strength of these effects such that thicker arrows represent stronger relations. Covariates (i.e., sex, age, education level, and marital status) and auto-regressive edges were excluded from the plot to ease visual interpretation. Node abbreviations: PSQI1 = subjective sleep quality; PSQI2 = sleep latency; PSQI3 = sleep duration; PSQI4 = sleep efficiency; PSQI5 = sleep disturbances; PSQI6 = use of sleep medication; PSQI7 = daytime dysfunction; K1 = tired out for no good reason; K2 = feel nervous; K3 = feel so nervous that nothing could calm you down; K4 = feel hopeless; K5 = restless or fidgety; K6 = feel so restless you could not sit still; K7 = feel depressed; K8 = feel that everything was an effort; K9 = feel so sad that nothing could cheer you up; K10 = feel worthless; MMSE1 = orientation; MMSE2 = registration; MMSE3 = attention and calculation; MMSE4 = recall; MMSE5 = language; MMSE6 = visuospatial. Figure 1. long description.

The CLPN using the total sample was plotted as a directed network (see Figure 1), in which arrows represent pairwise temporal relations between symptoms while controlling for covariates. Edge weights are presented in Supplementary Tables S3 and S4.

In the time intervals, the autoregressive edges were stronger than the cross-lagged edges (see Supplementary Figure S1), indicating that symptoms tend to predict themselves more strongly than they influence other symptoms across time points. Moreover, the analysis identified a total of 296 assessed cross-lagged edges (224 [75.68%] with OR > 1) at T1–T2 and 263 assessed cross-lagged edges (205[77.95%] with OR > 1) at T2–T3. In the first-time interval, the strongest cross-community associations were ‘Subjective sleep quality’ (PSQI1) → ‘Restless or fidgety’ (K5; edge weight = 0.138); ‘Use of sleep medication’ (PSQI6) → ‘Feel worthless’ (K10; edge weight = 0.101); ‘Use of sleep medication’ (PSQI6) → ‘Feel nervous’ (K2; edge weight = 0.100). In the second-time interval, the strongest cross-community associations revealed a shift in temporal dynamics: ‘Subjective sleep quality’ (PSQI1) → ‘Restless or fidgety’ (K5; edge weight = 0.129), followed by ‘Use of sleep medication’ (PSQI6) → ‘Orientation’ (MMSE1; edge weight = 0.105), ‘Restless or fidgety’ (K5) → ‘Orientation’ (MMSE1; edge weight = −0.100). Edge weights for other associations are provided in Supplementary Table S5. Additionally, within-community effects demonstrated the strongest associations, particularly within the cognitive function domain. The most robust cross-lagged effect was observed from ‘Registration’ (MMSE2) to ‘Recall’ (MMSE4; edge weight = 0.239), followed by ‘Registration’ (MMSE2) to ‘Orientation’ (MMSE1; edge weight = 0.232) at T1–T2. In the second time interval, within- community effects continued to dominate, with ‘Visuospatial’ (MMSE6) → ‘Language’ (MMSE5; edge weight = 0.242), followed by ‘Visuospatial’ (MMSE6) to ‘Attention and calculation’ (MMSE3; edge weight = 0.202).

**Figure 2.**
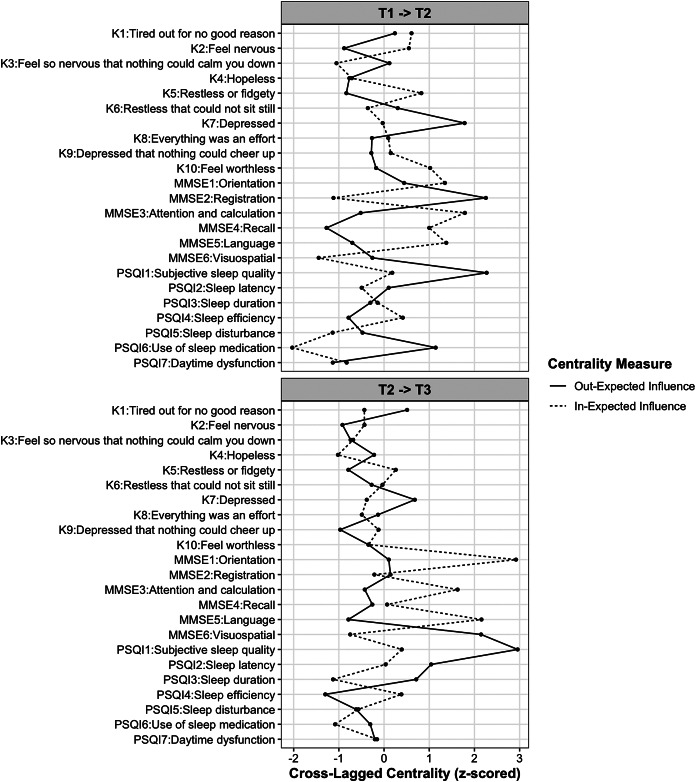
Standardized centrality estimates (out-EI and in-EI) for nodes in the cross-lagged panel network across time intervals. *Note: N* = 2,196. Larger values reflect greater centrality. Node abbreviations: PSQI1 = subjective sleep quality; PSQI2 = sleep latency; PSQI3 = sleep duration; PSQI4 = sleep efficiency; PSQI5 = sleep disturbances; PSQI6 = use of sleep medication; PSQI7 = daytime dysfunction; K1 = tired out for no good reason; K2 = feel nervous; K3 = feel so nervous that nothing could calm you down; K4 = feel hopeless; K5 = restless or fidgety; K6 = feel so restless you could not sit still; K7 = feel depressed; K8 = feel that everything was an effort; K9 = feel so sad that nothing could cheer you up; K10 = feel worthless; MMSE1 = orientation; MMSE2 = registration; MMSE3 = attention and calculation; MMSE4 = recall; MMSE5 = language; MMSE6 = visuospatial. Figure 2. long description.

**Figure 3.**
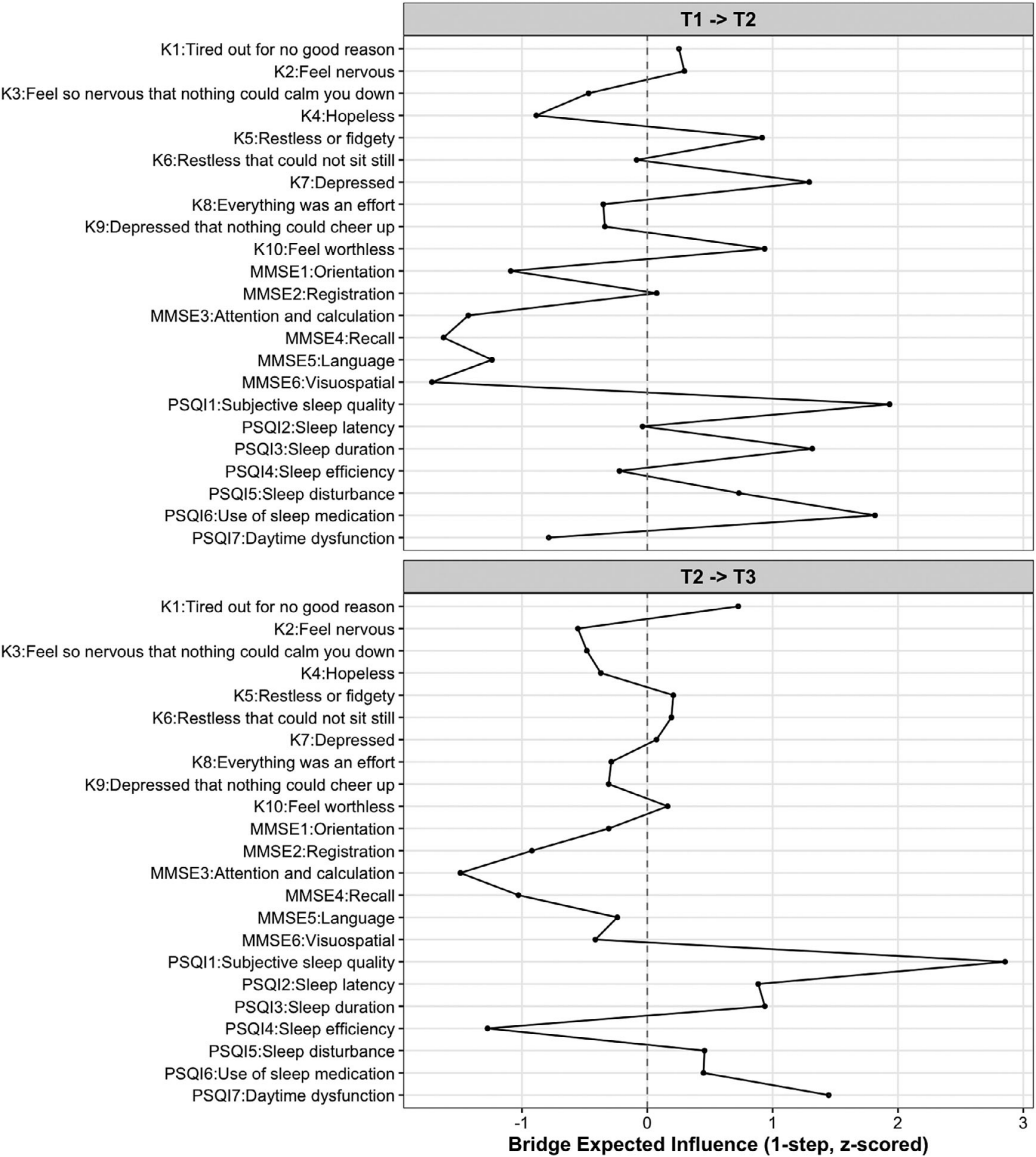
Bridge expected influence (1-step, z-scored) for nodes across time intervals (T1-T2 and T2-T3). *Note: N* = 2,196. Larger values indicate greater bridge influence. Figure 3. long description.

Standardized centrality estimates are plotted in Figure 2 and unstandardized coefficients are detailed in Supplementary Table S6. Out-EI measures revealed key predictive nodes across time intervals. ‘Subjective sleep quality’ (PSQI1) exhibited the highest Out-EI at both T1–T2 (0.960) and T2–T3 (1.007), establishing it as the most influential predictor node throughout the observation period. ‘Registration’ (MMSE2; 0.928) showed the second highest Out-EI at T1–T2, while ‘Visuospatial’ (MMSE6; 0.776) ranked second at T2–T3. In-EI identified the most influential nodes in the network. At T1–T2, the top nodes were ‘Attention and calculation’ (MMSE3; 0.611), ‘Language’ (MMSE5; 0.556), and ‘Orientation’ (MMSE1; 0.549). At T2-T3, ‘Orientation’ (MMSE1; 0.812) showed the highest In-EI, followed by ‘Language’ (MMSE5; 0.667) and ‘Attention and calculation’ (MMSE3; 0.567). BEI analysis (plotted in Figure 3) revealed that ‘Subjective sleep quality’ (PSQI1) presented the highest BEI value (BEI = 1.93 at T1–T2 and 2.85 at T2–T3). Node centrality differences are illustrated in Supplementary Figure S2.

### Sex-stratified CLPN results

**Figure 4.**
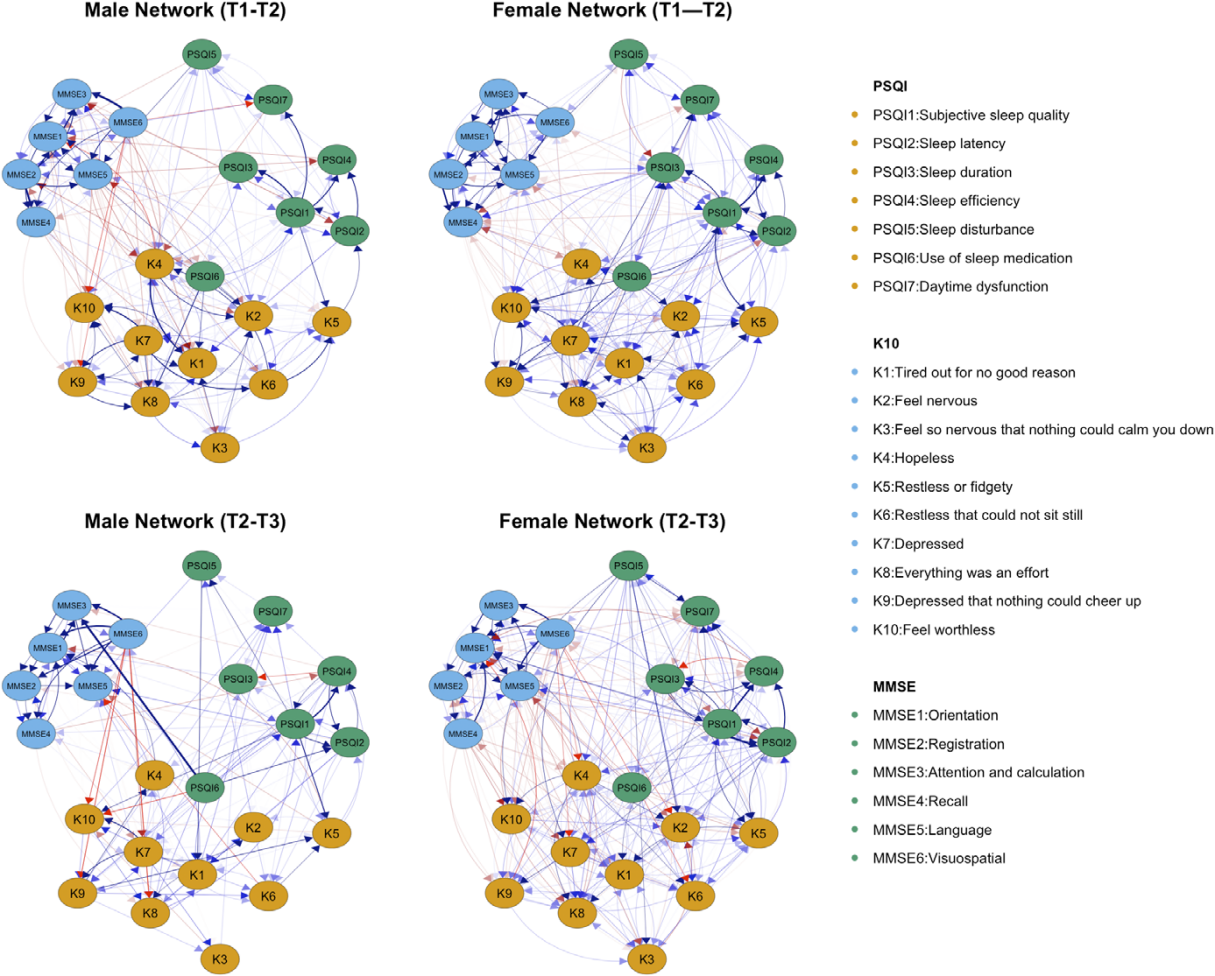
Sex-stratified cross-lagged panel networks showing temporal associations between sleep quality, cognitive function, and psychological distress. *Note: N* = 2,196. Blue arrows indicate positive coefficients and red arrows indicate negative coefficients. Arrow thickness represents the strength of these effects such that thicker arrows represent stronger relations. Covariates (i.e., sex, age, education level, marital status) and auto-regressive edges were excluded from the plot to ease visual interpretation. Figure 4. long description.

**Figure 5.**
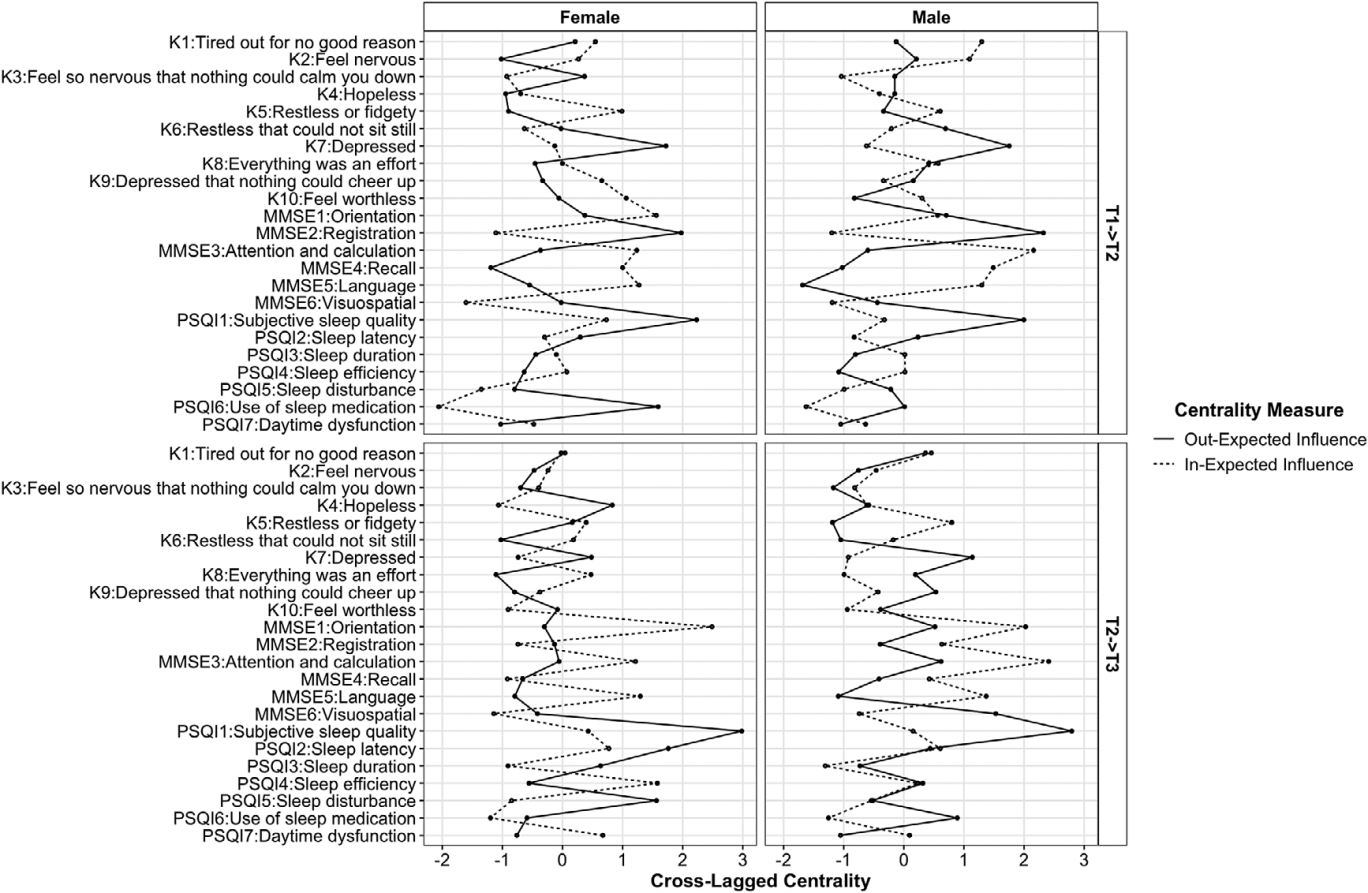
Standardized centrality estimates of sex-stratified cross-lagged panel networks for females and males. *Note*: Larger values reflect greater centrality. Figure 5. long description.

The network model was then constructed by sex. Figure 4 illustrates the sex-stratified CLPN, and Figure 5 shows the centrality estimates of sex-stratified networks. Networks between males and females demonstrated substantial structural similarity, particularly during T1–T2. The coefficient of similarity was strong (*r* = 0.657, *p* < 0.001), with high concordance in centrality measures: Out-EI (*r* = 0.755, *p* < 0.001) and In-EI (*r* = 0.819, *p* < 0.001). However, this similarity attenuated significantly during T2–T3, with the edge correlation decreasing to *r* = 0.516 (*p* < 0.001). Centrality concordance also weakened, particularly for Out-EI (*r* = 0.448, *p* = 0.032), while In-EI remained moderately correlated (*r* = 0.745, *p* < 0.001). Despite overall correlations, edge-level analyses revealed substantial sex differences. During T1–T2, the Jaccard index indicated moderate overlap (0.439), with 151 edges replicating with the same sign between networks, representing 65.9% of male network edges and 55.5% of female network edges. This overlap decreased during T2–T3 (Jaccard index = 0.352), with only 119 replicated edges representing 69.2% of male edges but merely 40.6% of female edges.

### Accuracy and stability of network parameters

For the total sample (Supplementary Figure S3) and sex-stratified networks (Supplementary Figure S4), bootstrapped CIs around the edge weights demonstrated adequate accuracy. The results of the stability test are shown in Supplementary Figures S5 and S6. Following established guidelines (Epskamp et al., 2018), where CS coefficients above 0.25 indicate acceptable stability and above 0.5 indicate good stability. CS coefficients exceeded 0.25 for all metrics, confirming sufficient stability. Female networks consistently demonstrated higher centrality stability across both time periods, particularly for In-EI measures.

## Discussions

This study employed CLPN analysis to examine the longitudinal relationships among sleep quality, cognitive function, and psychological distress in rural older adults using data from three waves. Overall, analysis of 2,196 rural older adults reveals that autoregressive effects were consistently stronger than cross-lagged effects across both time intervals, indicating that symptoms were more likely to predict themselves over time than to influence other symptoms. Centrality measures identified ‘Subjective sleep quality’ as the central hub within the networks, while cognitive components – particularly attention and calculation – emerged as the most vulnerable nodes. Sex-stratified analyses showed considerable structural similarities between males and females in the initial period, which diminished over time.

The CLPN network analysis revealed distinct temporal dynamics linking sleep quality, cognitive function, and psychological distress among rural older adults. The temporal architecture of these findings illuminates the mechanism through which *Scarring Theory* operates in late-life aging. Over time, PSQI demonstrated distinct predictive patterns across intervals: primarily influencing psychological distress (e.g., PSQI1 → K5, PSQI6 → K10) during T1–T2, whereas increasingly predicted cognitive functions (e.g., PSQI6 → MMSE1) during T2–T3. This shift suggests that sleep disorders might manifest as mood-related symptoms before progressively affecting cognitive functioning with advancing age (J. Wang, Chen, & Xue, 2024). Such an interpretation aligns with prior longitudinal network studies showing that persistent sleep problems are often associated with chronic health conditions, including comorbidities and cognitive impairment (Feng et al., 2025). Poor PSQI domains may affect aging through interconnected neurobiological mechanisms. From a biological mechanism, poor PSQI can contribute to psychological distress through dysregulation of the hypothalamic–pituitary–adrenal (HPA) axis and overactivation of the sympathetic nervous system. Concurrently, low sleep efficiency may lead to accelerated atrophy in the frontal, temporal, and parietal regions of the brain, which are crucial for memory and executive functions (Fjell et al., 2020; Stickgold & Walker, 2007). This temporal ordering is theoretically significant, as early adversity such as poor sleep leaves neurobiological scars that accumulate progressively across psychological and cognitive systems. Thus, early identification and intervention targeting sleep disturbances may help prevent the downstream cascade of psychological distress and cognitive decline in rural older adults.

The strong within-community associations among MMSE subscales suggest that cognitive functions operate as an interconnected system, in which impairments in one domain can cascade to others (Li, He, Hsu, & Li, 2025; Truong et al., 2024). For example, ‘Registration’ primarily drives impairments in ‘Recall’ (MMSE2 → MMSE4) and orientation (MMSE2 → MMSE1) in T1–T2, whereas ‘Visuospatial processing’ becomes the primary driver, influencing ‘Language’ functions (MMSE6 → MMSE5) and ‘Attention/calculation’ abilities (MMSE6 → MMSE3) in T2–T3. Although Henneges et al. observed that MMSE domains decline at different rates, they did not further examine the mutual influences among these domains (Henneges et al., 2016). Theoretical frameworks such as Cattell–Horn–Carroll (CHC) theory (McGrew, 2009) help explain these within-domain cognitive associations. During the T1–T2 period, impairment begins with basic memory processes: deficits in registration (MMSE2) prevent proper initial encoding of information, thereby hindering effective storage and retrieval, which subsequently leads to deterioration in recall (MMSE4) and orientation (MMSE1). As the condition progresses into T2–T3, the pattern shifts, with visuospatial processing (MMSE6) becoming the primary driver and affecting both language functions (MMSE5) and attention/calculation abilities (MMSE3). Rather than focusing on isolated cognitive domains, clinicians should adopt a network perspective that acknowledges the interconnected nature of cognitive functions. Early identification and targeted intervention for high-centrality cognitive symptoms may exert protective effects across the cognitive network.

The consistent identification of subjective sleep quality (PSQI1) as the node with the highest Out-EI and BEI across both time periods underscores its pivotal role as a temporal predictor within the aging-related symptom network. Compared with other sleep dimensions, the prominence of subjective sleep quality highlights its unique value in predicting perceived sleep satisfaction among rural older adults, reflecting sleep-related concerns that may drive downstream health outcomes (Buysse, 2014). This dominance over more objective sleep dimensions such as sleep duration and sleep efficiency may be partly explained by the broader health burden that subjective sleep complaints capture beyond sleep physiology alone (Vitiello, Larsen, & Moe, 2004). Prior study found that subjectively perceived poor sleep is linked to an increased risk of developing depression, and this might be related to slow wave sleep (McCarter et al., 2022). Additionally, sleep medication use (PSQI6) and sleep efficiency (PSQI4) also play certain predictive roles in psychological distress and cognitive function, aligning with existing studies (Blackwell et al., 2014; Cipriani, Bartoli, & Amanzio, 2021; Goldstein, Gaston, McGrath, & Jackson, 2020; Spira, Chen-Edinboro, Wu, & Yaffe, 2014). These findings highlight the need to move beyond conventional sleep duration metrics toward a more multidimensional assessment of sleep quality in aging research. Future longitudinal studies should investigate whether targeted sleep interventions implemented at earlier stages could help prevent or delay subsequent cognitive decline.

The identification of cognitive components – especially orientation, attention and calculation, and language functions – as highly influential In-EI nodes highlights their particular vulnerability within the aging symptom network. Orientation depends on the complex integration of vestibular–visual–proprioceptive systems, involving precise coordination among the vestibular nuclei, thalamus, parahippocampal gyrus, medial prefrontal cortex, and parietal regions (Denise, Harris, & Clément, 2022; Hanes & McCollum, 2006). This multi-system architecture renders spatial orientation particularly susceptible to sleep disturbances and psychological stress (Lupien, McEwen, Gunnar, & Heim, 2009; Ma et al., 2020). Attention and calculation rely on prefrontal executive networks, which have high metabolic demands and are among the first to decline with aging (Truong et al., 2024). These functions require intact working memory systems and precise frontoparietal connectivity, making them vulnerable to sleep-related neurophysiological disruptions (Dai et al., 2020). Language functions are affected more frequently, which may be related to the early appearance of language deficits in cognitive impairment (Verma & Howard, 2012; Woodward, 2013). Recognizing these three cognitive domains as central points of vulnerability within the aging network carries important clinical implications. Consequently, healthcare policies should prioritize comprehensive screening protocols targeting these specific cognitive domains.

Our analysis revealed that although the overall structure of male and female networks remained similar across both time periods, a temporal trend toward increasing differentiation was observed. The Jaccard similarity index decreased from 0.439 in T1–T2 to 0.352 in T2–T3, suggesting that sex-specific patterns may become more pronounced over time. This finding aligns with existing evidence indicating that sex differences in cognitive decline may become more pronounced at later stages of aging or in the context of more severe cognitive impairment (Arenaza-Urquijo et al., 2024). The observed decrease in network similarity further supports this interpretation. Longer follow-up durations or larger sample sizes could help uncover more substantial sex differences in aging populations. Additionally, methodological considerations inherent to network analysis may influence the detection of sex differences. While partial correlation networks offer insights into conditional relationships among symptoms, they may obscure sex differences that could be more apparent under other analytical frameworks.

These findings yield several important practical implications. First, the central role of subjective sleep quality suggests that sleep assessment and intervention should be prioritized within comprehensive geriatric care. Sleep hygiene education, cognitive-behavioral therapy for insomnia, and careful evaluation of sleep medication use may offer broad benefits across cognitive and psychological domains. Second, the network approach highlights the importance of addressing symptom interactions rather than treating isolated symptoms. Interventions targeting high-centrality nodes may lead to more efficient and sustained improvements across multiple domains. For example, improving sleep quality may simultaneously enhance cognitive function and alleviate psychological distress. Third, while our analysis did not detect significant sex differences at the current stage, the observed trend toward network differentiation over time suggests that sex-specific considerations may become increasingly relevant with advancing age.

## Limitations and future research

Several limitations should be acknowledged when interpreting these findings. First, the study employed different measurement approaches with distinct sources of bias. The PSQI and K10 are self-reported questionnaires susceptible to recall and response bias in older adults. The MMSE is a performance-based assessment conducted by an interviewer, which is influenced by the evaluator as well as the variability of the testing environment. Second, survivor bias may have influenced the T2–T3 analyses, as participants who remained in the study may represent a healthier subset of the original sample, potentially affecting the observed network structures and stability estimates. Third, the study did not account for important potential confounders such as medication use (beyond sleep medications), comorbid medical conditions, physical activity levels, and social support systems, which could significantly influence the observed symptom relationships. Finally, while CLPN analysis provides insights into temporal relationships, it cannot establish definitive causality, and unmeasured confounders or shorter-term bidirectional effects may influence the observed associations.

Future research should focus on several key directions. First, it is essential to explore the causal mechanisms behind the observed temporal associations using experimental or quasi-experimental designs. Second, researchers should develop and test interventions that are informed by network analysis, particularly those that target symptoms with high centrality. Finally, extending the observation period would allow for a more comprehensive understanding of long-term aging trajectories and the potential for non-linear changes in how symptoms relate to one another over time.

## Conclusions

This longitudinal network analysis, conducted among 2,196 rural older adults over 4 years, reveals dynamic interconnections among sleep quality, cognitive function, and psychological distress. Subjective sleep quality consistently emerged as the most influential predictor, with its primary influence shifting from psychological distress in earlier waves to cognitive functions in later stages. Interventions such as cognitive-behavioral therapy for insomnia, sleep hygiene education, and mindfulness-based sleep interventions should be prioritized in clinical practice and public health initiatives for rural older adults. Specific cognitive domains – orientation, attention and calculation, and language – were identified as particularly vulnerable, providing clear focus areas for early screening and intervention. While the overall network structure remained similar between males and females, a temporal trend toward increasing differentiation was observed. Taken together, this network approach provides a promising framework for developing targeted interventions and understanding the complex nature of aging-related symptoms in rural older adults.

## Supporting information

10.1017/S0033291726105182.sm001 Che et al. supplementary material Che et al. supplementary material

## Supplementary material

The supplementary material for this article can be found at http://doi.org/10.1017/S0033291726105182.

## Long descriptions

### Table 1. Long description

The table presents data for 13 variables across 15 columns. The columns are: Variables, Mean, Standard deviation, Sex, Education level, T 1 marital status, T 1 age, T 1 P S Q I, T 2 P S Q I, T 3 P S Q I, T 1 K 10, T 2 K 10, T 3 K 10, T 1 M M S E, and T 2 M M S E.

Key descriptive statistics (Mean, Standard Deviation):

* Sex: 1.66, 0.47

* Education level: 1.24, 0.53

* T 1 marital status: 0.76, 0.43

* T 1 age: 70.01, 5.89

* T 1 P S Q I: 7.68, 4.36

* T 2 P S Q I: 8.86, 4.17

* T 3 P S Q I: 6.59, 4.15

* T 1 K 10: 16.43, 7.27

* T 2 K 10: 17.92, 7.88

* T 3 K 10: 15.66, 6.75

* T 1 M M S E: 23.17, 4.92

* T 2 M M S E: 23.46, 5.40

* T 3 M M S E: 23.21, 5.23

Correlation highlights:

* T 1 P S Q I and T 2 P S Q I show a strong positive correlation of 0.683.

* T 1 K 10 and T 2 K 10 show a strong positive correlation of 0.694.

* M M S E scores across time points T 1, T 2, and T 3 show strong positive correlations ranging from 0.665 to 0.778.

* Age and Education level show negative correlations with M M S E scores across all time points.

* P S Q I and K 10 scores generally show positive correlations with each other and negative correlations with M M S E scores.

Navigate back to Table 1..

### Figure 1. Long description

A two-panel network diagram.

Panel 1 (Left): T 1 yields T 2 Network.

Panel 2 (Right): T 2 yields T 3 Network.

Both panels feature 23 nodes categorized into three color-coded clusters:

- Green nodes (P S Q I 1 to 7): Sleep quality indicators located primarily at the top and center-right.

- Blue nodes (M M S E 1 to 6): Cognitive function indicators located on the left side.

- Orange nodes (K 1 to 10): Psychological distress indicators located at the bottom and center-right.

Edges and Relationships:

- Blue arrows indicate positive coefficients; red arrows indicate negative coefficients.

- Arrow thickness represents effect strength.

- In both networks, dense blue arrows connect nodes within their own clusters (e.g., between M M S E nodes and between K nodes).

- Cross-cluster edges are visible, such as green P S Q I nodes pointing to orange K nodes and blue M M S E nodes.

- The T 2 yields T 3 network shows a similar structure to T 1 yields T 2 but with slight variations in arrow thickness, particularly a prominent red arrow from P S Q I 4 toward the M M S E cluster.

A legend on the right lists all node abbreviations:

- P S Q I: 1 Subjective sleep quality, 2 Sleep latency, 3 Sleep duration, 4 Sleep efficiency, 5 Sleep disturbance, 6 Use of sleep medication, 7 Daytime dysfunction.

- K 10: 1 Tired out, 2 Nervous, 3 So nervous nothing could calm, 4 Hopeless, 5 Restless or fidgety, 6 So restless could not sit still, 7 Depressed, 8 Everything was an effort, 9 So sad nothing could cheer up, 10 Worthless.

- M M S E: 1 Orientation, 2 Registration, 3 Attention and calculation, 4 Recall, 5 Language, 6 Visuospatial.

Navigate back to Figure 1..

### Figure 2. Long description

The graph consists of two vertically stacked panels. The x-axis represents Cross-Lagged Centrality z-scored values ranging from -2 to 3. The y-axis lists 23 nodes categorized as K 1 through K 10 (psychological distress), M M S E 1 through M M S E 6 (cognitive function), and P S Q I 1 through P S Q I 7 (sleep quality).

Top Panel (T 1 to T 2):

* Out-E I (solid line) shows high peaks at K 7 (Depressed), M M S E 2 (Registration), and P S Q I 1 (Subjective sleep quality), all reaching near or above 2.

* In-E I (dashed line) is generally more stable but shows a notable peak at M M S E 3 (Attention and calculation) near 2.

* P S Q I 6 (Use of sleep medication) shows the lowest Out-E I value, dropping below -1.5.

Bottom Panel (T 2 to T 3):

* Out-E I (solid line) shows its highest peak at P S Q I 1 (Subjective sleep quality) reaching 3, and another high point at M M S E 6 (Visuospatial) above 2.

* In-E I (dashed line) shows a significant spike at M M S E 1 (Orientation) reaching nearly 3, which is a sharp increase compared to the top panel.

* Sleep duration (P S Q I 3) and sleep efficiency (P S Q I 4) show low Out-E I values below -1.

Across both panels, P S Q I 1 consistently maintains high Out-Expected Influence, while cognitive nodes (M M S E) show high variability in In-Expected Influence between the two time intervals.

Navigate back to Figure 2..

### Figure 3. Long description

The figure consists of two vertically stacked panels. The x-axis for both is Bridge Expected Influence (1-step, z-scored) ranging from -1 to 3, with a dashed vertical line at 0. The y-axis lists 23 items categorized by K, M M S E, and P S Q I prefixes.

Top Panel (T 1 to T 2):

* K 1 to K 10: Data points fluctuate around the zero line. K 7 (Depressed) and K 10 (Feel worthless) show high positive influence, while K 4 (Hopeless) shows negative influence.

* M M S E 1 to M M S E 6: Most points are on the negative side, with M M S E 6 (Visuospatial) and M M S E 4 (Recall) showing the lowest values.

* P S Q I 1 to P S Q I 7: Shows high variability. P S Q I 1 (Subjective sleep quality), P S Q I 3 (Sleep duration), and P S Q I 6 (Use of sleep medication) show strong positive influence peaks.

Bottom Panel (T 2 to T 3):

* K 1 to K 10: The trend is more stabilized near zero compared to the first panel, with K 1 showing a slight positive shift.

* M M S E 1 to M M S E 6: Values remain largely negative, with M M S E 3 (Attention and calculation) being the most negative point.

* P S Q I 1 to P S Q I 7: P S Q I 1 (Subjective sleep quality) remains the highest positive influence peak, reaching nearly 3. P S Q I 4 (Sleep efficiency) shows a significant negative influence.

Navigate back to Figure 3..

### Figure 4. Long description

The figure consists of four circular network diagrams and a legend. The diagrams are arranged in a two-by-two grid: Male Network T 1 to T 2 (top-left), Female Network T 1 to T 2 (top-right), Male Network T 2 to T 3 (bottom-left), and Female Network T 2 to T 3 (bottom-right).

Each network contains three clusters of nodes:

* Green nodes (P S Q I 1 to 7): Represent sleep quality metrics, located at the top-right of each circle.

* Blue nodes (M M S E 1 to 6): Represent cognitive function, located at the top-left of each circle.

* Yellow nodes (K 1 to 10): Represent psychological distress, located at the bottom of each circle.

Edges between nodes are colored blue for positive coefficients and red for negative coefficients. Thicker lines indicate stronger temporal associations. In all networks, dense blue lines connect nodes within their own clusters, while thinner lines represent cross-lagged associations between different clusters. Notable thick blue lines appear between P S Q I 6 and M M S E 5 in the Male T 2 to T 3 network.

The legend on the right defines the labels:

* P S Q I: 1 Subjective sleep quality, 2 Sleep latency, 3 Sleep duration, 4 Sleep efficiency, 5 Sleep disturbance, 6 Use of sleep medication, 7 Daytime dysfunction.

* K 10: 1 Tired out for no good reason, 2 Feel nervous, 3 Feel so nervous that nothing could calm you down, 4 Hopeless, 5 Restless or fidgety, 6 Restless that could not sit still, 7 Depressed, 8 Everything was an effort, 9 Depressed that nothing could cheer up, 10 Feel worthless.

* M M S E: 1 Orientation, 2 Registration, 3 Attention and calculation, 4 Recall, 5 Language, 6 Visuospatial.

Navigate back to Figure 4..

### Figure 5. Long description

The graph consists of four panels. The top row represents the transition from T 1 to T 2, and the bottom row represents T 2 to T 3. The left column shows data for Female subjects, and the right column shows Male subjects. The x-axis represents Cross-Lagged Centrality ranging from -2 to 3. The y-axis lists 23 psychological and cognitive variables categorized by initialisms: K 1 through K 10 (Kessler Psychological Distress Scale), M M S E 1 through M M S E 6 (Mini-Mental State Examination), and P S Q I 1 through P S Q I 7 (Pittsburgh Sleep Quality Index).

Two data series are plotted in each panel: a solid line for Out-Expected Influence and a dashed line for In-Expected Influence.

* Top-Left (Female, T 1 to T 2): Out-Expected Influence peaks at P S Q I 1 (Subjective sleep quality) near 3.0. In-Expected Influence shows a significant peak at M M S E 1 (Orientation) near 1.5.

* Top-Right (Male, T 1 to T 2): Out-Expected Influence peaks at P S Q I 1 near 2.5. In-Expected Influence peaks at M M S E 3 (Attention and calculation) near 2.5.

* Bottom-Left (Female, T 2 to T 3): Out-Expected Influence shows a sharp peak at P S Q I 1 exceeding 3.0. In-Expected Influence peaks at M M S E 1 near 2.5.

* Bottom-Right (Male, T 2 to T 3): Out-Expected Influence peaks at P S Q I 1 near 2.8. In-Expected Influence peaks at M M S E 3 near 2.5.

Across all panels, P S Q I 1 consistently shows high Out-Expected Influence, while M M S E variables show higher In-Expected Influence compared to the K series.

Navigate back to Figure 5..
