# Supplementary material for: Longitudinal relations between sleep quality, psychological distress, and cognitive function among rural older adults: a cross-lagged network analysis
Source: Psychol Med. 2026 Jul 28;56:e237. doi: 10.1017/S0033291726105182 (PMC13439242; doi:10.1017/S0033291726105182)
Supplement: Supplementary file 1 [file S0033291726105182sup001.docx]

**Supplementary Materials**

Additional supporting information can be found in the online version of this article.

[Table S1. Domains, individual items and maximum score of items of the MMSE	2](#_Toc227053698)

[Table S2. Node labels and descriptive statistics at different time points.	3](#_Toc227053699)

[Table S3. Odds Ratios of cross-lagged (T1-T2) network association in total sample	4](#_Toc227053700)

[Table S4. Odds Ratios of cross-lagged (T2-T3) network association in total sample	5](#_Toc227053701)

[Table S5. Edge Weight for Each Cross-lagged Network Association in total sample.	6](#_Toc227053702)

[Table S6. Specific values of Out-EI and In-EI in total sample.	9](#_Toc227053703)

[Figure S1. Autoregressive edges for each symptom in total sample.	10](#_Toc227053704)

[Figure S2. Out-EI and in-EI centrality difference tests in the total sample.	11](#_Toc227053705)

[Figure S3. Edge weight accuracy for the network of the total sample.	12](#_Toc227053706)

[Figure S4. Edge Weight Accuracy for Sex-stratified Networks	13](#_Toc227053707)

[Figure S5. Stability of the centrality measures for the network of the total sample.	14](#_Toc227053708)

[Figure S6. Stability of the Centrality Measures for Sex-stratified Networks.	15](#_Toc227053709)

# Table S1. Domains, individual items and maximum score of items of the MMSE

| **Domains** | **Individual items** | **Max item score** | **Domain Total** |
| --- | --- | --- | --- |
| Orientation | 1. What is the year? Season? Date? Day? Month? | 5 | 10 |
|  | 2. Where are we now? State? County? Town/city? Hospital? Floor? | 5 |  |
| Registration | 3. Listen to the names of three objects and then recall them | 3 | 3 |
| Attention | 4. Subtract 7 from 100 and keep subtracting 7 from each new number for five times | 5 | 5 |
| Recall | 5. Recall three objects read out earlier | 3 | 3 |
| Language | 6. What is this called? | 2 | 8 |
|  | 7. Repeat a phrase: “Forty-four stone lions (a Chinese tongue twister)” | 1 |  |
|  | 8. Take this piece of paper in your right hand, fold it in half using both hands, and put the piece of paper on your thigh. | 3 |  |
|  | 9. Read the words on this page and then do what it says | 1 |  |
|  | 10. Write or say any complete sentence | 1 |  |
| Visuospatial | 11. Copy this design (pentagons) | 1 | 1 |

# Table S2. Node labels and descriptive statistics at different time points.

| **Label** | **Item** | **Score Range** | **T1(2019)** | | | **T2(2020)** | | | **T3(2022)** | | |
| --- | --- | --- | --- | --- | --- | --- | --- | --- | --- | --- | --- |
|  |  |  | **M ± SD** | **Skewness** | **Kurtosis** | **M ± SD** | **Skewness** | **Kurtosis** | **M ± SD** | **Skewness** | **Kurtosis** |
| PSQI1 | Subjective sleep quality | 0-3 | 1.05±0.90 | 0.50 | 2.44 | 1.20±0.86 | 0.34 | 2.48 | 1.08±0.90 | 0.48 | 2.46 |
| PSQI2 | Sleep latency |  | 1.46±1.17 | 0.06 | 1.53 | 1.71±1.15 | -0.22 | 1.60 | 1.41±1.25 | 0.14 | 1.39 |
| PSQI3 | Sleep duration |  | 1.42±0.98 | -0.08 | 1.95 | 1.69±0.95 | -0.36 | 2.23 | 0.53±0.79 | 1.33 | 3.84 |
| PSQI4 | Sleep efficiency |  | 1.24±1.23 | 0.30 | 1.46 | 1.67±1.20 | -0.25 | 1.52 | 1.24±1.19 | 0.32 | 1.56 |
| PSQI5 | Sleep disturbance |  | 1.14±0.61 | 0.32 | 3.53 | 1.30±0.58 | 0.43 | 3.21 | 1.21±0.57 | 0.40 | 3.53 |
| PSQI6 | Use of sleep medication |  | 0.12±0.52 | 4.66 | 24.06 | 0.13±0.56 | 4.38 | 21.29 | 0.12±0.53 | 4.51 | 22.62 |
| PSQI7 | Daytime dysfunction |  | 1.25±1.12 | 0.24 | 1.65 | 1.16±1.10 | 0.40 | 1.79 | 1±1.1 | 0.62 | 1.97 |
| K1 | Tired out for no good reason | 1-5 | 1.96±1.18 | 0.82 | 2.42 | 2.06±1.20 | 0.69 | 2.23 | 1.77±1.04 | 1.02 | 2.75 |
| K2 | Feel nervous |  | 1.68±0.99 | 1.22 | 3.36 | 1.79±1.04 | 1.01 | 2.89 | 1.49±0.84 | 1.54 | 4.24 |
| K3 | Feel so nervous that nothing could calm you down |  | 1.50±0.88 | 1.75 | 5.35 | 1.61±0.95 | 1.42 | 4.12 | 1.39±0.77 | 1.96 | 5.94 |
| K4 | Hopeless |  | 1.46±0.86 | 1.92 | 6.09 | 1.56±0.94 | 1.60 | 4.67 | 1.43±0.83 | 1.87 | 5.51 |
| K5 | Restless or fidgety |  | 1.75±1.05 | 1.14 | 3.21 | 1.91±1.11 | 0.84 | 2.50 | 1.68±0.97 | 1.17 | 3.21 |
| K6 | Restless that could not sit still |  | 1.59±0.93 | 1.45 | 4.16 | 1.68±0.98 | 1.23 | 3.44 | 1.53±0.87 | 1.49 | 4.11 |
| K7 | Depressed |  | 1.55±0.92 | 1.58 | 4.68 | 1.73±1.00 | 1.18 | 3.45 | 1.52±0.87 | 1.56 | 4.51 |
| K8 | Everything was an effort |  | 1.62±0.98 | 1.39 | 3.88 | 1.85±1.07 | 0.93 | 2.71 | 1.62±0.99 | 1.42 | 3.94 |
| K9 | Depressed that nothing could cheer up |  | 1.59±0.98 | 1.61 | 4.67 | 1.79±1.07 | 1.17 | 3.37 | 1.55±0.92 | 1.53 | 4.29 |
| K10 | Feel worthless |  | 1.73±1.14 | 1.38 | 3.70 | 1.94±1.20 | 0.94 | 2.64 | 1.68±1.05 | 1.28 | 3.37 |
| MMSE1 | Orientation | 0-10 | 8.66±1.70 | -1.55 | 5.50 | 8.52±1.89 | -1.62 | 5.67 | 8.46±1.78 | -1.40 | 4.91 |
| MMSE2 | Registration | 0-3 | 2.68±0.79 | -2.51 | 8.16 | 2.58±0.82 | -2.07 | 6.40 | 2.48±0.85 | -1.64 | 4.83 |
| MMSE3 | Attention and calculation | 0-5 | 2.62±1.88 | 0.02 | 1.45 | 2.82±1.91 | -0.19 | 1.46 | 2.79±1.86 | -0.22 | 1.55 |
| MMSE4 | Recall | 0-3 | 1.87±1.20 | -0.52 | 1.70 | 2.10±1.13 | -0.88 | 2.27 | 2.15±1.07 | -0.96 | 2.54 |
| MMSE5 | Language | 0-8 | 6.95±1.02 | -1.42 | 5.98 | 7.03±1.12 | -1.73 | 8.10 | 6.93±1.13 | -1.31 | 5.42 |
| MMSE6 | Visuospatial | 0-1 | 0.39±0.49 | 0.45 | 1.20 | 0.41±0.49 | 0.35 | 1.12 | 0.41±0.49 | 0.36 | 1.13 |

# Table S3. Odds Ratios of cross-lagged (T1-T2) network association in total sample

|  | **PSQI1** | **PSQI2** | **PSQI3** | **PSQI4** | **PSQI5** | **PSQI6** | **PSQI7** | **K1** | **K2** | **K3** | **K4** | **K5** | **K6** | **K7** | **K8** | **K9** | **K10** | **MMSE1** | **MMSE2** | **MMSE3** | **MMSE4** | **MMSE5** | **MMSE6** |
| --- | --- | --- | --- | --- | --- | --- | --- | --- | --- | --- | --- | --- | --- | --- | --- | --- | --- | --- | --- | --- | --- | --- | --- |
| **PSQI1** | 1.42 | 1.12 | 1.17 | 1.22 | 1.04 | 1.04 | 1.08 | 1.03 | 1.06 | 1.03 | 1.00 | 1.15 | 1.05 | 1.05 | 1.00 | 1.01 | 1.00 | 1.00 | 1.01 | 1.00 | 1.00 | 0.99 | 0.97 |
| **PSQI2** | 1.08 | 1.46 | 1.05 | 1.12 | 1.01 | 1.01 | 1.04 | 1.06 | 1.03 | 1.01 | 1.02 | 1.04 | 1.03 | 1.00 | 1.02 | 1.01 | 1.00 | 1.00 | 0.98 | 0.97 | 0.97 | 0.98 | 0.99 |
| **PSQI3** | 1.05 | 1.00 | 1.39 | 1.00 | 1.01 | 1.00 | 1.00 | 1.00 | 1.00 | 1.00 | 1.02 | 1.00 | 1.00 | 1.04 | 1.03 | 1.01 | 1.04 | 1.00 | 1.00 | 1.00 | 1.03 | 1.03 | 1.02 |
| **PSQI4** | 1.02 | 1.10 | 1.00 | 1.39 | 1.01 | 1.00 | 1.03 | 1.00 | 1.00 | 1.00 | 1.00 | 1.00 | 0.99 | 0.99 | 1.00 | 1.00 | 1.03 | 0.98 | 1.00 | 1.00 | 1.00 | 1.00 | 0.99 |
| **PSQI5** | 1.01 | 1.00 | 0.95 | 1.00 | 1.35 | 1.00 | 1.07 | 1.00 | 1.00 | 1.00 | 1.00 | 1.00 | 1.02 | 1.02 | 1.00 | 1.00 | 1.02 | 1.00 | 1.00 | 1.00 | 1.03 | 1.05 | 1.05 |
| **PSQI6** | 1.11 | 1.06 | 1.04 | 1.00 | 1.05 | 1.76 | 1.01 | 1.03 | 1.10 | 1.00 | 1.02 | 1.03 | 1.02 | 1.06 | 1.10 | 1.09 | 1.11 | 1.00 | 1.00 | 0.99 | 0.96 | 0.95 | 0.98 |
| **PSQI7** | 1.03 | 1.00 | 1.01 | 1.00 | 1.01 | 1.00 | 1.30 | 1.01 | 1.00 | 1.00 | 1.00 | 1.00 | 1.00 | 0.98 | 0.98 | 1.00 | 1.00 | 1.00 | 1.00 | 1.00 | 1.00 | 1.01 | 1.00 |
| **K1** | 1.00 | 1.02 | 1.00 | 1.03 | 1.00 | 1.00 | 1.01 | 1.39 | 1.02 | 1.01 | 1.03 | 1.04 | 1.00 | 1.07 | 1.05 | 1.08 | 1.06 | 1.00 | 1.00 | 1.00 | 1.00 | 1.03 | 1.00 |
| **K2** | 1.00 | 1.00 | 1.00 | 0.99 | 1.00 | 1.00 | 1.00 | 1.03 | 1.48 | 1.00 | 1.01 | 1.00 | 1.03 | 1.01 | 1.04 | 1.00 | 1.00 | 1.00 | 1.00 | 1.00 | 1.00 | 1.00 | 0.99 |
| **K3** | 1.00 | 1.00 | 1.00 | 1.00 | 1.00 | 1.01 | 1.00 | 1.09 | 1.05 | 1.60 | 1.00 | 1.06 | 1.04 | 1.04 | 1.07 | 1.03 | 1.01 | 1.01 | 1.00 | 1.00 | 1.00 | 1.00 | 1.00 |
| **K4** | 1.00 | 1.00 | 1.00 | 1.00 | 1.00 | 1.00 | 1.00 | 1.04 | 1.04 | 1.00 | 1.55 | 1.01 | 1.04 | 1.03 | 1.00 | 1.00 | 1.06 | 1.00 | 0.99 | 1.00 | 1.00 | 0.97 | 0.97 |
| **K5** | 1.04 | 1.05 | 1.01 | 1.00 | 1.00 | 1.00 | 1.00 | 1.03 | 1.01 | 1.01 | 1.00 | 1.41 | 1.00 | 1.00 | 1.00 | 1.00 | 1.00 | 1.00 | 1.00 | 1.00 | 1.00 | 0.97 | 1.01 |
| **K6** | 1.00 | 1.00 | 1.00 | 1.00 | 1.00 | 1.00 | 1.00 | 1.06 | 1.06 | 1.04 | 1.07 | 1.09 | 1.51 | 1.03 | 1.02 | 1.04 | 1.00 | 1.00 | 1.00 | 1.00 | 1.00 | 1.00 | 1.02 |
| **K7** | 1.04 | 1.00 | 1.03 | 1.00 | 1.00 | 1.00 | 1.06 | 1.05 | 1.00 | 1.05 | 1.07 | 1.07 | 1.06 | 1.64 | 1.10 | 1.11 | 1.15 | 1.00 | 1.04 | 1.00 | 1.01 | 1.02 | 1.02 |
| **K8** | 1.00 | 1.00 | 1.00 | 1.00 | 1.00 | 1.00 | 1.00 | 1.05 | 1.09 | 1.05 | 1.02 | 1.00 | 1.04 | 1.02 | 1.46 | 1.02 | 1.00 | 1.00 | 0.98 | 1.00 | 1.00 | 1.04 | 0.99 |
| **K9** | 1.00 | 1.00 | 1.00 | 1.00 | 1.00 | 1.00 | 1.00 | 1.04 | 1.00 | 1.00 | 1.00 | 1.01 | 1.01 | 1.03 | 1.06 | 1.45 | 1.12 | 1.00 | 1.01 | 1.00 | 1.00 | 1.00 | 1.01 |
| **K10** | 1.02 | 1.00 | 1.04 | 1.05 | 1.02 | 1.00 | 1.00 | 1.01 | 1.00 | 1.00 | 1.02 | 1.03 | 1.01 | 1.04 | 1.00 | 1.07 | 1.41 | 1.00 | 1.00 | 1.00 | 1.00 | 1.00 | 1.00 |
| **MMSE1** | 1.00 | 1.00 | 1.00 | 1.00 | 1.00 | 1.00 | 1.00 | 1.00 | 1.00 | 1.00 | 0.99 | 1.00 | 0.98 | 0.99 | 0.99 | 1.00 | 1.00 | 1.75 | 1.06 | 1.22 | 1.13 | 1.12 | 1.02 |
| **MMSE2** | 1.00 | 0.99 | 1.03 | 1.02 | 1.01 | 1.00 | 1.00 | 1.00 | 1.00 | 1.00 | 1.00 | 1.00 | 1.03 | 1.02 | 1.00 | 1.00 | 1.02 | 1.26 | 1.54 | 1.16 | 1.27 | 1.18 | 1.02 |
| **MMSE3** | 1.00 | 1.00 | 1.00 | 1.00 | 1.00 | 1.00 | 1.00 | 0.99 | 0.98 | 0.98 | 1.00 | 0.99 | 0.99 | 0.98 | 0.99 | 1.00 | 1.00 | 1.11 | 1.03 | 1.55 | 1.04 | 1.09 | 1.04 |
| **MMSE4** | 1.00 | 0.98 | 1.00 | 1.00 | 1.00 | 1.00 | 0.99 | 0.97 | 1.00 | 1.00 | 0.98 | 1.00 | 0.98 | 0.98 | 0.97 | 0.98 | 0.99 | 1.04 | 1.02 | 1.06 | 1.23 | 1.02 | 1.03 |
| **MMSE5** | 1.00 | 1.00 | 1.00 | 0.97 | 1.00 | 1.00 | 0.99 | 0.98 | 1.00 | 1.00 | 1.00 | 0.99 | 0.99 | 1.00 | 1.00 | 1.00 | 0.96 | 1.08 | 1.06 | 1.06 | 1.08 | 1.30 | 1.01 |
| **MMSE6** | 1.00 | 0.98 | 1.01 | 1.03 | 1.00 | 0.98 | 0.95 | 0.99 | 1.00 | 1.00 | 1.00 | 1.00 | 1.00 | 0.99 | 0.95 | 0.94 | 0.98 | 1.11 | 1.00 | 1.21 | 1.01 | 1.16 | 1.24 |

*Note.* Adjacency matrix of the T1→T2 cross-lagged panel network in total. Independent variables (i.e., predictors) are in columns, and dependent variables are in rows. Autoregressive edges are presented along the diagonal.

# Table S4. Odds Ratios of cross-lagged (T2-T3) network association in total sample

|  | **PSQI1** | **PSQI2** | **PSQI3** | **PSQI4** | **PSQI5** | **PSQI6** | **PSQI7** | **K1** | **K2** | **K3** | **K4** | **K5** | **K6** | **K7** | **K8** | **K9** | **K10** | **MMSE1** | **MMSE2** | **MMSE3** | **MMSE4** | **MMSE5** | **MMSE6** |
| --- | --- | --- | --- | --- | --- | --- | --- | --- | --- | --- | --- | --- | --- | --- | --- | --- | --- | --- | --- | --- | --- | --- | --- |
| **PSQI1** | 1.17 | 1.19 | 1.07 | 1.19 | 1.04 | 1.03 | 1.03 | 1.05 | 1.03 | 1.00 | 1.00 | 1.14 | 1.08 | 1.02 | 1.04 | 1.04 | 1.02 | 1.09 | 1.00 | 1.00 | 1.00 | 1.00 | 1.00 |
| **PSQI2** | 1.07 | 1.23 | 1.05 | 1.11 | 1.03 | 1.00 | 1.04 | 1.05 | 1.02 | 1.02 | 1.00 | 1.05 | 1.03 | 1.03 | 1.04 | 1.00 | 1.02 | 1.00 | 1.00 | 1.00 | 1.00 | 1.00 | 1.00 |
| **PSQI3** | 1.11 | 1.04 | 1.25 | 1.05 | 1.02 | 1.00 | 1.00 | 1.00 | 1.00 | 1.00 | 1.00 | 1.02 | 1.00 | 1.01 | 1.00 | 1.00 | 1.00 | 1.08 | 1.00 | 1.06 | 1.01 | 1.07 | 1.00 |
| **PSQI4** | 1.04 | 1.06 | 0.91 | 1.11 | 1.00 | 1.00 | 1.04 | 1.00 | 1.00 | 1.00 | 1.02 | 1.03 | 1.00 | 1.03 | 1.01 | 1.03 | 1.01 | 0.91 | 0.98 | 0.91 | 0.99 | 0.96 | 1.00 |
| **PSQI5** | 1.00 | 1.00 | 1.00 | 1.00 | 1.06 | 1.00 | 1.00 | 1.00 | 1.00 | 1.00 | 1.00 | 1.00 | 1.00 | 1.00 | 1.00 | 1.00 | 1.00 | 1.02 | 1.00 | 1.03 | 1.00 | 1.06 | 1.01 |
| **PSQI6** | 1.04 | 1.00 | 1.00 | 1.00 | 1.00 | 1.33 | 1.00 | 1.00 | 1.00 | 1.00 | 1.00 | 1.02 | 1.05 | 1.00 | 1.00 | 1.00 | 1.00 | 1.11 | 1.00 | 1.01 | 0.98 | 0.98 | 1.00 |
| **PSQI7** | 1.00 | 1.00 | 1.00 | 1.00 | 1.00 | 1.00 | 1.05 | 1.00 | 1.00 | 1.00 | 1.00 | 1.00 | 1.00 | 1.00 | 1.00 | 1.02 | 1.01 | 1.04 | 1.00 | 1.06 | 1.02 | 1.06 | 1.00 |
| **K1** | 1.04 | 1.01 | 1.02 | 1.04 | 1.01 | 1.00 | 1.06 | 1.06 | 1.01 | 1.00 | 1.03 | 1.02 | 1.00 | 1.03 | 1.06 | 1.05 | 1.03 | 1.00 | 1.00 | 1.00 | 1.00 | 1.00 | 1.00 |
| **K2** | 1.00 | 1.00 | 0.98 | 1.00 | 1.00 | 1.00 | 1.00 | 1.00 | 1.04 | 1.02 | 1.00 | 1.00 | 1.03 | 1.01 | 1.00 | 1.00 | 1.00 | 1.00 | 1.00 | 1.00 | 1.00 | 1.00 | 1.00 |
| **K3** | 1.00 | 1.00 | 1.00 | 1.00 | 1.02 | 1.00 | 1.03 | 1.01 | 1.00 | 1.00 | 1.00 | 1.00 | 1.03 | 1.01 | 1.00 | 1.00 | 1.00 | 1.01 | 1.00 | 0.99 | 1.00 | 0.98 | 1.00 |
| **K4** | 1.00 | 1.00 | 1.00 | 1.00 | 1.00 | 1.00 | 1.05 | 1.00 | 1.03 | 1.00 | 1.06 | 1.00 | 1.03 | 1.02 | 1.03 | 1.01 | 1.03 | 1.02 | 1.00 | 1.00 | 1.00 | 1.00 | 1.01 |
| **K5** | 1.00 | 1.01 | 1.01 | 1.00 | 1.00 | 1.02 | 1.00 | 1.02 | 1.00 | 1.01 | 1.02 | 1.02 | 1.03 | 1.00 | 1.00 | 1.03 | 1.02 | 0.91 | 1.00 | 1.00 | 1.00 | 0.98 | 1.00 |
| **K6** | 1.00 | 1.00 | 1.00 | 1.01 | 1.03 | 1.00 | 1.02 | 1.02 | 1.07 | 1.04 | 1.01 | 1.05 | 1.04 | 1.00 | 1.00 | 1.00 | 1.01 | 1.00 | 1.00 | 1.00 | 0.99 | 0.98 | 1.00 |
| **K7** | 1.00 | 1.00 | 1.02 | 1.00 | 1.00 | 1.00 | 1.00 | 1.04 | 1.03 | 1.02 | 1.01 | 1.05 | 1.03 | 1.06 | 1.06 | 1.08 | 1.08 | 1.00 | 1.00 | 1.00 | 1.03 | 1.00 | 1.00 |
| **K8** | 1.05 | 1.00 | 1.00 | 1.00 | 1.00 | 1.00 | 1.00 | 1.04 | 1.02 | 1.03 | 1.03 | 1.00 | 1.00 | 1.03 | 1.06 | 1.03 | 1.01 | 1.00 | 1.00 | 1.00 | 1.00 | 1.00 | 1.00 |
| **K9** | 1.00 | 1.00 | 1.00 | 1.00 | 1.02 | 1.00 | 1.00 | 1.00 | 1.00 | 1.00 | 1.00 | 1.00 | 1.00 | 1.00 | 1.00 | 1.00 | 1.01 | 1.00 | 1.00 | 0.99 | 1.00 | 1.00 | 1.00 |
| **K10** | 1.00 | 1.00 | 0.98 | 1.00 | 1.00 | 1.01 | 1.02 | 1.00 | 1.00 | 1.00 | 1.00 | 1.00 | 1.00 | 1.03 | 1.02 | 1.01 | 1.03 | 1.06 | 1.00 | 1.01 | 1.01 | 1.04 | 1.00 |
| **MMSE1** | 1.00 | 1.00 | 1.01 | 0.99 | 1.00 | 1.00 | 0.99 | 0.97 | 1.00 | 1.00 | 0.99 | 0.98 | 0.99 | 0.99 | 0.95 | 0.97 | 0.98 | 1.39 | 1.09 | 1.16 | 1.10 | 1.13 | 1.02 |
| **MMSE2** | 1.00 | 1.00 | 1.00 | 1.00 | 1.00 | 1.00 | 1.00 | 1.00 | 1.00 | 1.00 | 0.98 | 1.00 | 1.00 | 1.00 | 0.99 | 1.00 | 1.00 | 1.16 | 1.04 | 1.07 | 1.03 | 1.05 | 1.04 |
| **MMSE3** | 0.99 | 0.98 | 1.00 | 0.98 | 1.00 | 1.00 | 0.97 | 0.99 | 0.99 | 0.98 | 1.00 | 1.00 | 0.98 | 0.99 | 0.98 | 0.99 | 0.99 | 1.17 | 1.03 | 1.48 | 1.03 | 1.10 | 1.03 |
| **MMSE4** | 1.00 | 1.00 | 1.00 | 1.00 | 1.00 | 1.00 | 0.99 | 1.00 | 1.00 | 1.00 | 1.00 | 0.97 | 1.00 | 1.00 | 1.00 | 1.00 | 0.98 | 1.10 | 1.06 | 1.06 | 1.14 | 1.05 | 1.01 |
| **MMSE5** | 1.00 | 1.00 | 1.00 | 1.00 | 1.00 | 1.00 | 1.00 | 1.00 | 1.00 | 1.00 | 0.98 | 0.99 | 0.99 | 1.00 | 1.00 | 1.00 | 0.99 | 1.04 | 1.00 | 1.05 | 1.02 | 1.13 | 1.00 |
| **MMSE6** | 1.00 | 1.00 | 1.00 | 1.00 | 1.00 | 1.00 | 1.00 | 1.00 | 1.00 | 1.00 | 1.00 | 1.01 | 1.00 | 1.00 | 1.00 | 1.00 | 1.00 | 1.17 | 1.07 | 1.22 | 1.10 | 1.27 | 1.21 |

*Note.* Adjacency matrix of the T2→T3 cross-lagged panel network in total. Independent variables (i.e., predictors) are in columns, and dependent variables are in rows. Autoregressive edges are presented along the diagonal.

# Table S5. Edge Weight for Each Cross-lagged Network Association in total sample.

|  | **T1→T2** | | **T2→T3** | |
| --- | --- | --- | --- | --- |
|  | **Edge** | **Edge Weight** | **Edge** | **Edge Weight** |
| 1 | Registration (MMSE2) → Recall (MMSE4) | 0.239 | Visuospatial (MMSE6) → Language (MMSE5) | 0.242 |
| 2 | Registration (MMSE2) → Orientation (MMSE1) | 0.232 | Visuospatial (MMSE6) → Attention and calculation (MMSE3) | 0.202 |
| 3 | Subjective sleep quality (PSQI1) → Sleep efficiency (PSQI4) | 0.203 | Subjective sleep quality (PSQI1) → Sleep efficiency (PSQI4) | 0.178 |
| 4 | Orientation (MMSE1) → Attention and calculation (MMSE3) | 0.199 | Subjective sleep quality (PSQI1) → Sleep latency (PSQI2) | 0.174 |
| 5 | Visuospatial (MMSE6) → Attention and calculation (MMSE3) | 0.192 | Attention and calculation (MMSE3) → Orientation (MMSE1) | 0.155 |
| 6 | Registration (MMSE2) → Language (MMSE5) | 0.168 | Visuospatial (MMSE6) → Orientation (MMSE1) | 0.154 |
| 7 | Subjective sleep quality (PSQI1) → Sleep duration (PSQI3) | 0.156 | Orientation (MMSE1) → Attention and calculation (MMSE3) | 0.150 |
| 8 | Registration (MMSE2) → Attention and calculation (MMSE3) | 0.148 | Registration (MMSE2) → Orientation (MMSE1) | 0.145 |
| 9 | Visuospatial (MMSE6) → Language (MMSE5) | 0.147 | Subjective sleep quality (PSQI1) → Restless or fidgety (K5) | 0.129 |
| 10 | Depressed (K7) → Feel worthless (K10) | 0.139 | Orientation (MMSE1) → Language (MMSE5) | 0.120 |
| 11 | Subjective sleep quality (PSQI1) → Restless or fidgety (K5) | 0.138 | Sleep duration (PSQI3) → Subjective sleep quality (PSQI1) | 0.109 |
| 12 | Orientation (MMSE1) → Recall (MMSE4) | 0.123 | Use of sleep medication (PSQI6) → Orientation (MMSE1) | 0.105 |
| 13 | Orientation (MMSE1) → Language (MMSE5) | 0.116 | Sleep latency (PSQI2) → Sleep efficiency (PSQI4) | 0.103 |
| 14 | Depressed that nothing could cheer up (K9) → Feel worthless (K10) | 0.114 | Restless or fidgety (K5) → Orientation (MMSE1) | -0.100 |
| 15 | Subjective sleep quality (PSQI1) → Sleep latency (PSQI2) | 0.114 | Orientation (MMSE1) → Recall (MMSE4) | 0.099 |
| 16 | Sleep latency (PSQI2) → Sleep efficiency (PSQI4) | 0.110 | Recall (MMSE4) → Orientation (MMSE1) | 0.099 |
| 17 | Depressed (K7) → Depressed that nothing could cheer up (K9) | 0.109 | Attention and calculation (MMSE3) → Language (MMSE5) | 0.098 |
| 18 | Attention and calculation (MMSE3) → Orientation (MMSE1) | 0.106 | Sleep efficiency (PSQI4) → Attention and calculation (MMSE3) | -0.098 |
| 19 | Visuospatial (MMSE6) → Orientation (MMSE1) | 0.105 | Sleep efficiency (PSQI4) → Orientation (MMSE1) | -0.095 |
| 20 | Use of sleep medication (PSQI6) → Subjective sleep quality (PSQI1) | 0.101 | Visuospatial (MMSE6) → Recall (MMSE4) | 0.095 |
| 21 | Use of sleep medication (PSQI6) → Feel worthless (K10) | 0.101 | Sleep efficiency (PSQI4) → Sleep duration (PSQI3) | -0.093 |
| 22 | Use of sleep medication (PSQI6) → Feel nervous (K2) | 0.100 | Subjective sleep quality (PSQI1) → Orientation (MMSE1) | 0.087 |
| 23 | Use of sleep medication (PSQI6) → Everything was an effort (K8) | 0.100 | Orientation (MMSE1) → Registration (MMSE2) | 0.083 |
| 24 | Sleep efficiency (PSQI4) → Sleep latency (PSQI2) | 0.093 | Depressed (K7) → Feel worthless (K10) | 0.080 |
| 25 | Depressed (K7) → Everything was an effort (K8) | 0.093 | Sleep duration (PSQI3) → Orientation (MMSE1) | 0.079 |
| 26 | Feel so nervous that nothing could calm you down (K3) → Tired out for no good reason (K1) | 0.090 | Depressed (K7) → Depressed that nothing could cheer up (K9) | 0.078 |
| 27 | Use of sleep medication (PSQI6) → Depressed that nothing could cheer up (K9) | 0.086 | Subjective sleep quality (PSQI1) → Restless that could not sit still (K6) | 0.073 |
| 28 | Restless that could not sit still (K6) → Restless or fidgety (K5) | 0.086 | Sleep duration (PSQI3) → Language (MMSE5) | 0.072 |
| 29 | Everything was an effort (K8) → Feel nervous (K2) | 0.082 | Visuospatial (MMSE6) → Registration (MMSE2) | 0.070 |
| 30 | Attention and calculation (MMSE3) → Language (MMSE5) | 0.082 | Sleep latency (PSQI2) → Subjective sleep quality (PSQI1) | 0.069 |
| 31 | Language (MMSE5) → Orientation (MMSE1) | 0.081 | Registration (MMSE2) → Attention and calculation (MMSE3) | 0.068 |
| 32 | Subjective sleep quality (PSQI1) → Daytime dysfunction (PSQI7) | 0.078 | Restless that could not sit still (K6) → Feel nervous (K2) | 0.068 |
| 33 | Sleep latency (PSQI2) → Subjective sleep quality (PSQI1) | 0.076 | Subjective sleep quality (PSQI1) → Sleep duration (PSQI3) | 0.065 |
| 34 | Language (MMSE5) → Recall (MMSE4) | 0.074 | Tired out for no good reason (K1) → Daytime dysfunction (PSQI7) | 0.062 |
| 35 | Tired out for no good reason (K1) → Depressed that nothing could cheer up (K9) | 0.073 | Daytime dysfunction (PSQI7) → Attention and calculation (MMSE3) | 0.062 |
| 36 | Sleep disturbance (PSQI5) → Daytime dysfunction (PSQI7) | 0.071 | Depressed (K7) → Everything was an effort (K8) | 0.061 |
| 37 | Restless that could not sit still (K6) → Hopeless (K4) | 0.070 | Sleep efficiency (PSQI4) → Sleep latency (PSQI2) | 0.059 |
| 38 | Feel worthless (K10) → Depressed that nothing could cheer up (K9) | 0.069 | Tired out for no good reason (K1) → Everything was an effort (K8) | 0.057 |
| 39 | Depressed (K7) → Restless or fidgety (K5) | 0.065 | Recall (MMSE4) → Attention and calculation (MMSE3) | 0.057 |
| 40 | Tired out for no good reason (K1) → Depressed (K7) | 0.065 | Feel worthless (K10) → Orientation (MMSE1) | 0.057 |
| 41 | Depressed (K7) → Hopeless (K4) | 0.064 | Recall (MMSE4) → Registration (MMSE2) | 0.057 |
| 42 | Feel so nervous that nothing could calm you down (K3) → Everything was an effort (K8) | 0.063 | Sleep disturbance (PSQI5) → Language (MMSE5) | 0.057 |
| 43 | Depressed (K7) → Restless that could not sit still (K6) | 0.062 | Daytime dysfunction (PSQI7) → Language (MMSE5) | 0.056 |
| 44 | Language (MMSE5) → Registration (MMSE2) | 0.062 | Sleep duration (PSQI3) → Attention and calculation (MMSE3) | 0.056 |
| 45 | Restless that could not sit still (K6) → Tired out for no good reason (K1) | 0.061 | Subjective sleep quality (PSQI1) → Tired out for no good reason (K1) | 0.053 |
| 46 | Restless that could not sit still (K6) → Feel nervous (K2) | 0.061 | Orientation (MMSE1) → Everything was an effort (K8) | -0.053 |
| 47 | Language (MMSE5) → Attention and calculation (MMSE3) | 0.061 | Everything was an effort (K8) → Subjective sleep quality (PSQI1) | 0.053 |
| 48 | Visuospatial (MMSE6) → Depressed that nothing could cheer up (K9) | -0.060 | Registration (MMSE2) → Language (MMSE5) | 0.052 |
| 49 | Hopeless (K4) → Feel worthless (K10) | 0.060 | Sleep latency (PSQI2) → Restless or fidgety (K5) | 0.050 |
| 50 | Orientation (MMSE1) → Registration (MMSE2) | 0.059 | Use of sleep medication (PSQI6) → Restless that could not sit still (K6) | 0.050 |

*Note. N*=2196. Only the top 50 edges (ranked by absolute coefficient magnitude) are displayed.

# Table S6. Specific values of Out-EI and In-EI in total sample.

| **Node** | **T1→T2** | | **T2→T3** | |
| --- | --- | --- | --- | --- |
|  | **in-EI** | **out-EI** | **in-EI** | **out-EI** |
| **PSQI1** | 0.387 | 0.960 | 0.349 | 1.007 |
| **PSQI2** | 0.272 | 0.382 | 0.269 | 0.542 |
| **PSQI3** | 0.327 | 0.277 | 0.042 | 0.454 |
| **PSQI4** | 0.407 | 0.137 | 0.335 | -0.082 |
| **PSQI5** | 0.176 | 0.225 | 0.163 | 0.115 |
| **PSQI6** | 0.029 | 0.664 | 0.059 | 0.192 |
| **PSQI7** | 0.221 | 0.041 | 0.243 | 0.223 |
| **K1** | 0.456 | 0.428 | 0.187 | 0.408 |
| **K2** | 0.445 | 0.112 | 0.188 | 0.037 |
| **K3** | 0.189 | 0.392 | 0.139 | 0.084 |
| **K4** | 0.243 | 0.142 | 0.070 | 0.220 |
| **K5** | 0.486 | 0.126 | 0.320 | 0.065 |
| **K6** | 0.301 | 0.443 | 0.267 | 0.203 |
| **K7** | 0.355 | 0.848 | 0.200 | 0.449 |
| **K8** | 0.366 | 0.284 | 0.173 | 0.242 |
| **K9** | 0.375 | 0.278 | 0.249 | 0.024 |
| **K10** | 0.511 | 0.312 | 0.206 | 0.186 |
| **MMSE1** | 0.549 | 0.458 | 0.812 | 0.278 |
| **MMSE2** | 0.176 | 0.928 | 0.229 | 0.303 |
| **MMSE3** | 0.611 | 0.208 | 0.567 | 0.147 |
| **MMSE4** | 0.491 | -0.003 | 0.283 | 0.202 |
| **MMSE5** | 0.556 | 0.155 | 0.667 | 0.070 |
| **MMSE6** | 0.123 | 0.252 | 0.127 | 0.776 |

# Figure S1. Autoregressive edges for each symptom in total sample.

# Figure S2. Out-EI and in-EI centrality difference tests in the total sample.

*Note. N*=2196.*Black boxes* indicate edges that significantly differ in edge weights (*p*<.05), and *gray boxes* indicate edges that are not significantly different.

# Figure S3. Edge weight accuracy for the network of the total sample.

*Note. N*=2196. The *red line* indicates the edge weight in the estimated sample network and the *gray area* indicates the 95% bootstrapped confidence intervals. Each *horizontal line* represents one edge of the network, ordered from the edge with the highest edge-weight to the edge with the lowest edge-weight.

# Figure S4. Edge Weight Accuracy for Sex-stratified Networks

*Note. N*=2196. (A) Network for males in T1-T2; (B) Network for females in T1-T2. (C) Network for males in T2-T3; (D) Network for females in T2-T3. The *red line* indicates the edge weight in the estimated sample network and the *gray area* indicates the 95% bootstrapped confidence intervals. Each *horizontal line* represents one edge of the network, ordered from the edge with the highest edge-weight to the edge with the lowest edge-weight.

# Figure S5. Stability of the centrality measures for the network of the total sample.

*Note. N*=2196. Average correlations between centrality indices of network sampled with persons dropped and the original sample. *Lines* indicate the means and *areas* indicate the range from the 2.5th quantile to the 97.5 quantile.

# Figure S6. Stability of the Centrality Measures for Sex-stratified Networks.

*Note. N*=2196. (A) Network for males in T1-T2; (B) Network for females in T1-T2. (C) Network for males in T2-T3; (D) Network for females in T2-T3.
